# 

**Published:** 1900-10

**Authors:** 


### BOOKS RECEIVED.

The Student's Medical Dictionary. Including all the Words and Phrases Generally Used in Medicine, with their Proper Pronunciation and Definitions. Based on Recent Medical Literature. By George M. Gould, A. M., M. D., Author of "An Illustrated Dictionary of Medicine, Biology, and Allied Sciences," etc. Large 12mo. Eleventh edition, enlarged. Illustrated. Philadelphia: P. Blakiston's Son & Co. 1900. [Price, $2.50.]

The Medical Directory of New York, New Jersey and Connecticut. Published by the New York State Medical Association. Volume II. 1900. [Price, $2.50.]

Brain in Relation to Mind. By J. Sanderson Christison, M. D., Author of "Crime and Criminals," etc. Duodecimo, pp. 142. Second edition. Chicago: The Meng Publishing Co. 1900. [Price, $1.25.]

Perfect Eyes. A Complete and Comprehensive Treatise on the Organ of Vision. Duodecimo, pp. 71. Illustrated. Sixth edition. New York: Benjamin F. Stephens, Publishers. 1900.

Medical Diseases of Infancy and Childhood. By Dawson Williams, M. D., Physician to the East London Hospital for Children. New (2d) edition. Specially revised for America by F. S. Churchill, A. B., M. D., Instructor in Diseases of Children, Rush Medical College. Octavo, pp. 538, 52 illustrations and 2 colored plates. Philadelphia and New York: Lea Brothers & Co. 1900. [Cloth, $3.50 net.]

Progressive Medicine. Vol. III., September, 1900. A Quarterly Digest of Advances, Discoveries and Improvements in the Medical and Surgical Sciences. Edited by Hobart Amory Hare, M. D., Professor of Therapeutics and Materia Medica in Jefferson Medical College of Philadelphia. Octavo, pp. 408, with 14 engravings. Issued quarterly. Philadelphia and New York: Lea Brothers & Co. 1900. [Price, $10.00 per year ]

Manual of Diseases of the Eye. For Students and General Practitioners. By Charles H. May, M. D., Chief of Clinic and Instructor in Ophthalmology, Eye Department, College of Physicians and Surgeons, New York. Illustrated. Duodecimo, pp. 419 New York: William Wood & Co. 1900.

Transactions of the Royal Dublin Society, Vol. VII., Parts 2–7; Proceedings, Vol. IX, Parts 1 and 2; Economic Proceedings, Vol. I., Parts 1 and 2. Index, 1877 to 1898, inclusive. Dr. A. H. Foord, F. G. S., Editor.

A Treatise on Mental Diseases. Based Upon the Lecture Course at the Johns Hopkins University, 1899, and Designed for the Use of Practitioners and Students of Medicine. By Henry J. Berkley, M. D., Clinical Professor of Psychiatry, Johns Hopkins University, etc. Octavo, pp. xiv—601. Illustrated. New York: D. Appleton & Company. 1900.

A Treatise on Diseases of the Nose and Throat. By Ernest L. Shurley, M. D., Vice-president and Professor of Laryngology and Clinical Medicine, Detroit College of Medicine; Laryngologist and late Chief of Staff, Harper Hospital; Consulting Laryngologist, St. Mary's Hospital, etc. Octavo, pp xvii.—744. Illustrated. New York. D. Appleton & Company. 1900.

Transactions of the American Surgical Association. Volume XVIII. Session, held May 1–3, 1900. Edited by De Forest Willard, M. D., Ph. D., Recorder of the Association. Philadelphia: Wm. J Dornan, Printer.

Transactions of the Medical Society of the State of New York. For the Year 1900. Ninety-fourth annual session, held at Albany, January 30, 31 and February 1, 1900. Frederic C. Curtis, M. D., Secretary. Philadelphia: Wm. J. Dornan, Printer.

Twenty-sixth Annual Report of the Secretary of the State Board of Health of the State of Michigan for the Fiscal Year Ending June 30, 1898. Henry B. Baker, M. D., Secretary. Lansing: State Printers. 1900.

Report of the Mortality Records of the Mutual Life Insurance of New York. For fifty-six years—1843 to 1898. By Elias J. Marsh, M. D., and Granville M. White, M. D., Medical Directors. Published by the Company. 1900.

